# Astragaloside IV ameliorates cognitive impairment after traumatic brain injury with involvement of NMDAR-related signaling

**DOI:** 10.3389/fphar.2026.1925675

**Published:** 2026-10-29

**Authors:** Chenglang Jiang, Weiqi Shen, Xin Liu, Yuhang Hui, Boru Hou

**Affiliations:** 1 The Second Hospital & Clinical Medical School, Lanzhou University, Lanzhou, Gansu, China; 2 Key Laboratory of Biomonitoring and Bioremediation for Environmental Pollution, School of Life Sciences, Lanzhou University, Lanzhou, Gansu, China; 3 Academician Workstation of the Second Hospital & Clinical Medical School, Lanzhou University, Lanzhou, Gansu, China

**Keywords:** astragaloside IV, cognitive impairment, dendritic structure, N-methyl-D-aspartate receptor, synapse-associated proteins, traumatic brain injury

## Abstract

Traumatic brain injury (TBI) can cause persistent cognitive impairment accompanied by neuronal and synaptic damage. Astragaloside IV (AS-IV) has neuroprotective properties, but the signaling processes associated with its effects after TBI remain incompletely understood. Adult male mice underwent controlled cortical impact and received intraperitoneal AS-IV during a 28-day post-injury period, with GM1 included as a positive control. Behavioral assessments, immunofluorescence, Golgi-Cox staining, transmission electron microscopy, and immunoblotting were combined with NMDAR antagonist co-treatment and exploratory docking, molecular dynamics, and cellular thermal shift analyses. AS-IV treatment improved cognitive performance and was associated with preservation of dendritic and synaptic structure, increased NMDAR subunit expression, and changes in ERK1/2, CaMKII, and CREB phosphorylation and synapse-associated proteins. The 40 mg/kg dose showed greater effects than 80 mg/kg for several outcomes. Co-treatment with MK-801 or TCN-201 attenuated multiple AS-IV-associated effects, supporting involvement of NMDAR-related signaling. Computational predictions and NMDAR2A thermal stabilization were consistent with possible receptor engagement but did not establish direct binding, binding affinity, or receptor activation. Collectively, these findings suggest that modulation of NMDAR-related signaling alterations may contribute to the cognitive and synaptic structural and molecular effects associated with AS-IV after TBI. However, the present data do not establish direct NMDAR binding or activation, *in vivo* target occupancy, or NMDAR as the exclusive or primary molecular target of AS-IV.

## Graphical Abstract

**Figure:**
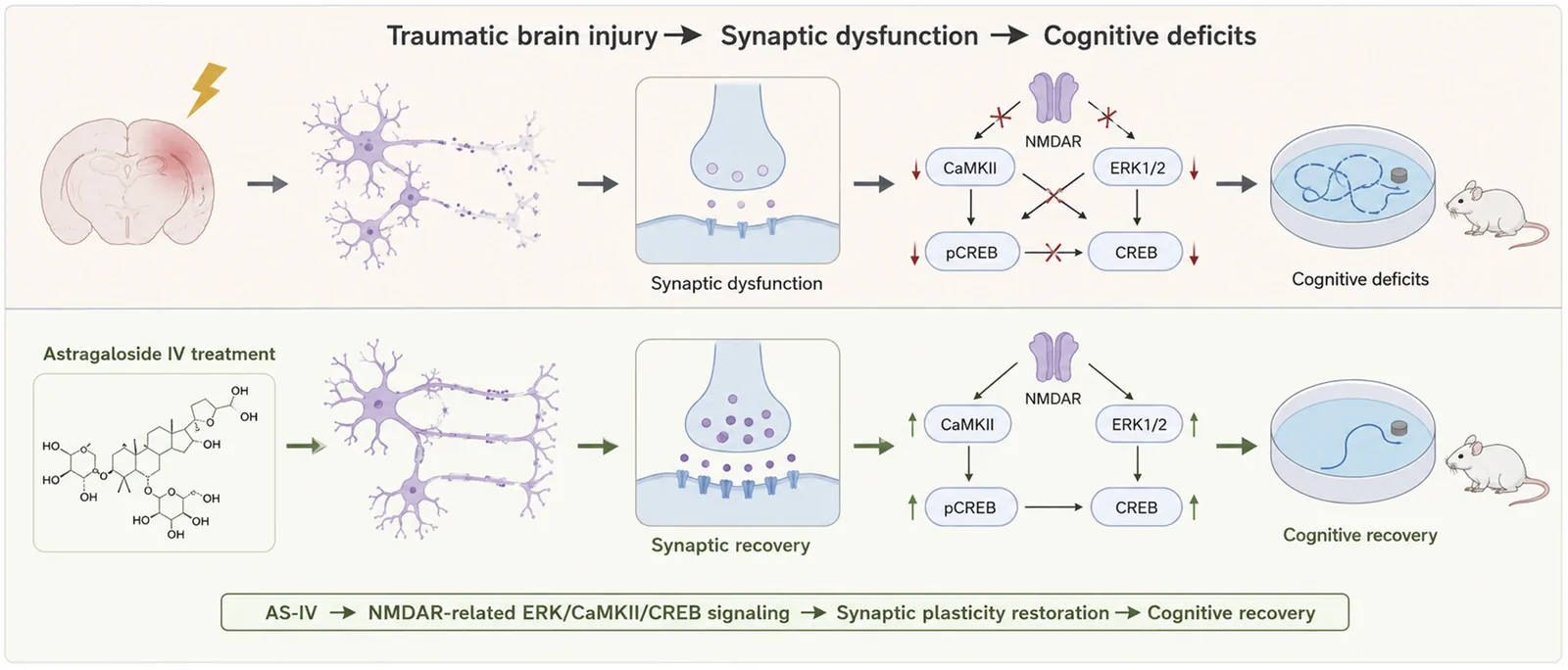
Schematic illustration of the proposed effects of astragaloside IV (AS-IV) on synaptic dysfunction and cognitive deficits after traumatic brain injury (TBI).

## 1 Introduction

Traumatic brain injury (TBI) is a widespread global health issue with over 50 million incident cases annually worldwide (Manley et al., 2025). Notably, TBI cases account for over 18% of the global burden in China (Jiang et al., 2019). As a persistent public health challenge worldwide, severe TBI results in 33% mortality, and 33% of survivors either experience severe disability or vegetative states. Meanwhile, survivors and mild cases often develop headaches, depression, and behavioral, cognitive, or emotional impairments (Jebbia et al., 2024; McCauley et al., 2014), along with a heightened risk of neurodegenerative disorders, including Alzheimer’s, Parkinson’s, and epilepsy (Rostowsky et al., 2021; de Freitas Cardoso et al., 2019). TBI pathophysiology includes primary and secondary injuries. Primary injury causes blood-brain barrier disruption, releases inflammatory mediators, causes free radical overload, excites glutamate release, and leads to secondary neurodegeneration (Maas et al., 2022). Secondary injury involves complex cellular processes and biochemical cascades initiated minutes to days after trauma (Lu et al., 2026). Furthermore, these secondary processes significantly worsen primary damage and are a leading cause of in-hospital TBI death (Panza et al., 2022). It has been shown that secondary injury impairs synapse-associated molecular profiles, including markers such as activity-regulated cytoskeleton-associated protein (ARC), brain-derived neurotrophic factor (BDNF), synaptophysin, and postsynaptic density-95 (PSD-95) (Witkowski et al., 2019). Dysfunctional plasticity results in abnormal synaptic connections that impair cognition and memory (Jamjoom et al., 2021). Furthermore, functional recovery after TBI depends on neural regeneration and the strengthening of synaptic plasticity (Xiong et al., 2019). *In vivo* studies of TBI show synapse loss and increased elimination through long-term depression (LTD). Moderate-to-severe TBI causes a rapid increase followed by a decrease of calcium/calmodulin-dependent kinase II (CaMKII) in the hippocampus, along with reduced expression of synaptic proteins such as NMDARs (Svirsky et al., 2024; Shohami and Biegon, 2014). Within synapses, NMDARs mediate transmission; their activation leads to postsynaptic depolarization and induces long-term potentiation (LTP), which enhances learning (Mony and Paoletti, 2023). Although not the only plasticity regulator, CaMKII is the most crucial protein in excitatory synapses and plays a central role in synaptic plasticity for learning and memory (Yasuda et al., 2022). Despite advances in TBI treatment, strategies for lasting neuroprotection and synaptic plasticity repair remain difficult. Additionally, BDNF is crucial for reducing synaptic loss and cognitive deficits (Wurzelmann et al., 2017). Studies indicate that CREB is phosphorylated at Ser133 by NMDAR-mediated Ca^2+^ influx to control BDNF transcription, forming a key pathway for synaptic plasticity and cognition (Hardingham et al., 2001; Barco et al., 2002). Therefore, targeting this pathway has therapeutic potential for post-TBI neural repair and cognitive improvement.

Astragaloside IV (AS-IV), a neuroprotective saponin derived from Astragalus membranaceus, has broad applications against neurodegeneration (Costa et al., 2019). Previous studies show AS-IV improves axonal regeneration, rebuilds synapses, and promotes neurogenesis in the hippocampus. In Alzheimer’s models, AS-IV reduces Aβ production through PPARγ-associated inhibition of β-site APP-cleaving enzyme 1 (BACE1) (Wang et al., 2017). Also, in ischemic stroke, which shares similar pathology with TBI, injury cascades lead to neuronal loss and worsening cognitive decline, resulting in vascular cognitive impairment (Amanollahi et al., 2023). Previous studies indicate that AS-IV reduces injury through multiple mechanisms, including JAK2/STAT3-associated protection against cerebral ischemia-reperfusion injury (Xu et al., 2020) and reduced interleukin-17 expression with improved cognition and hippocampal neurogenesis in stroke mice (Sun et al., 2020). Additionally, studies of radiation-induced neuronal injury implicate BDNF/TrkB signaling in AS-IV-associated protection (Liu et al., 2023; Liu X. et al., 2024), while ERK-related effects have been reported in radiation-induced neuronal senescence (Ding et al., 2025). AS-IV has also been studied in stress-associated cognitive deficits in relation to gut microbial metabolism (Liu Q. et al., 2024). Therefore, the evidence of AS-IV’s effectiveness against secondary pathological change suggests its potential for TBI treatment (Ding et al., 2025; Liang et al., 2023). However, current TBI treatment research is limited, and available therapies mainly focus on managing the primary injury. Given the complexity of post-TBI pathology and its multiform causes, single-target drugs are unlikely to address all neurological and cognitive issues (Reddi et al., 2022). AS-IV, a natural compound that targets multiple pathways, is a promising neuroprotective and regenerative agent. Still, there is little evidence about its ability to shorten recovery time. Thus, we propose that AS-IV offers neuroprotective effects and reduces post-TBI cognitive deficits. In our research, we observed changes in cortical neuron structure, dendritic spine density, and MAP-2 expression in TBI mice with AS-IV treatment, combined with modifications in NMDAR subunit expression and CaMKII activity, which accompanied improved learning and memory in AS-IV-treated TBI mice.

## 2 Materials and methods

### 2.1 Animals

Adult male C57BL/6 mice (8–10 weeks old, 22–25 g) were obtained from the Laboratory Animal Center of Lanzhou University (Lanzhou, China). The experiments were approved by the Animal Care and Use Committee of the Second Hospital of Lanzhou University (approval no. D2022-085). Animals were maintained under controlled temperature and humidity with a 12-h light/12-h dark cycle and had free access to food and water. Male mice were selected at the study-design stage with the aim of reducing potential variability associated with fluctuations in ovarian hormone levels across the estrous cycle.

### 2.2 Experimental groups and drug preparation

The study comprised two experiments. Experiment I examined AS-IV-associated behavioral and tissue changes over 28 days after CCI (Figure 1B). Mice were randomly allocated to five groups (nine mice per group): sham + vehicle, TBI + vehicle, TBI + AS-IV (40 mg/kg), TBI + AS-IV (80 mg/kg), and TBI + GM1 (2 mg/kg). GM1 was included as a positive control because of its established role in neuronal membrane biology and neuronal remodeling (Benady et al., 2018). AS-IV and GM1 were administered intraperitoneally every other day after CCI during the 28-day observation period. Experiment II included five additional groups (nine mice per group): sham + vehicle, TBI + vehicle, TBI + AS-IV (40 mg/kg), TBI + AS-IV + TCN-201, and TBI + AS-IV + MK-801. AS-IV followed the same regimen as in Experiment I. TCN-201, a GluN2A-selective negative allosteric modulator, and MK-801, a non-subtype-selective NMDAR channel blocker (MedChemExpress, USA), were administered intraperitoneally at 0.1 mg/kg in PBS every 3 days during post-injury days 14–28.

**FIGURE 1:**
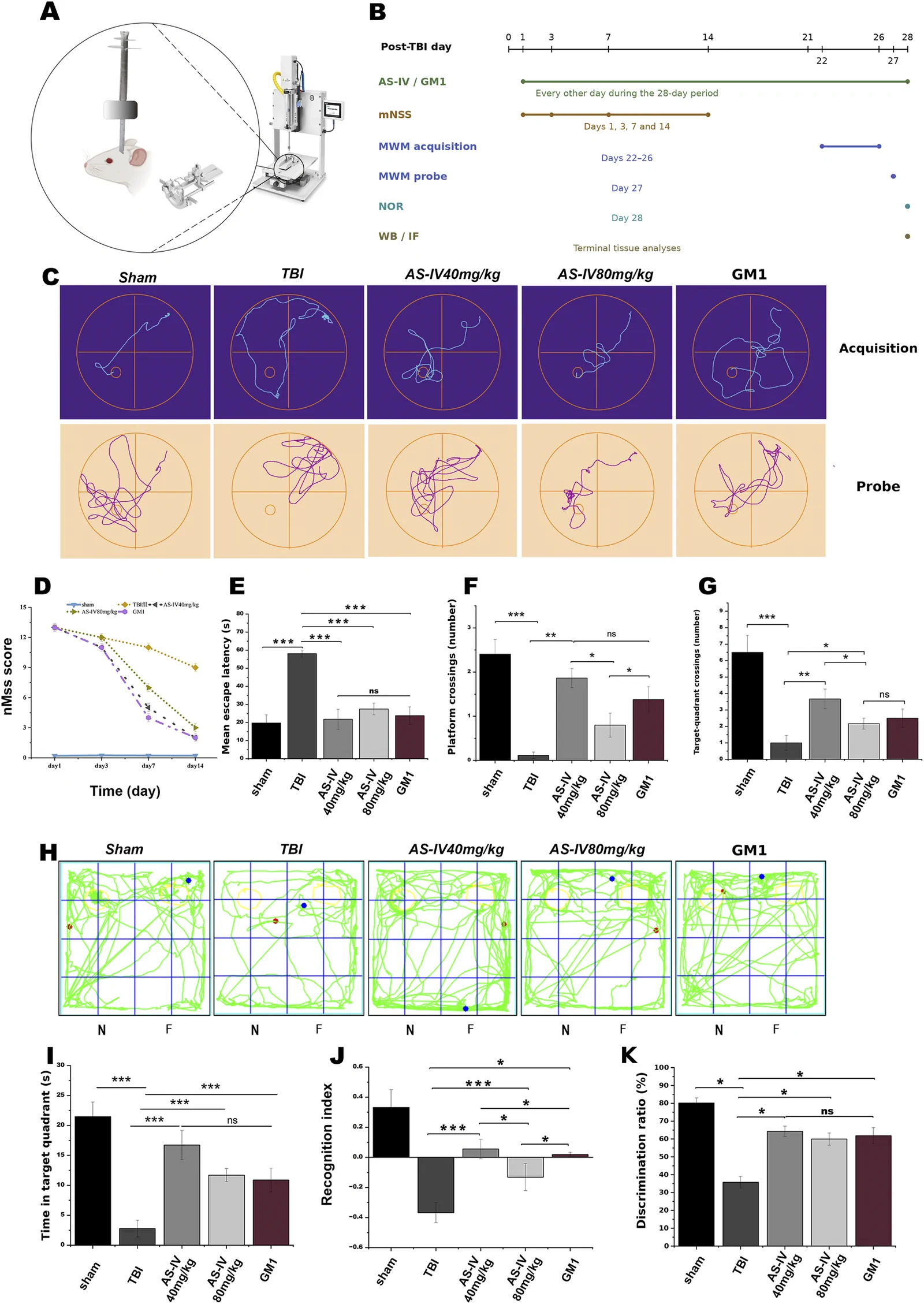
AS-IV attenuates neurological and behavioral impairments after TBI. **(A)** Schematic illustration of the mouse TBI model established by controlled cortical impact (RWD 68099II). **(B)** Timeline of Experiment I. **(C)** Representative MWM acquisition trajectories during post-injury days 22–26. **(D)** Changes in the modified neurological severity score (mNSS) on days 1, 3, 7, and 14 after TBI. **(E)** Mean escape latency averaged across the five-day MWM acquisition period. **(F)** Number of crossings over the former platform location during the MWM spatial-probe test. **(G)** Number of target-quadrant crossings during the spatial-probe test. **(H)** Representative NOR trajectories on day 28 after TBI. **(I)** Time spent in the target zone during the MWM probe trial. **(J)** Recognition index and **(K)** discrimination ratio in the NOR test. Data are mean ± SEM; n = 6 mice per group. *p < 0.05, **p < 0.01, ***p < 0.001; ns, not significant. Sham and TBI groups received the matched drug-free vehicle.

AS-IV was purchased from Shanghai Yuanye Bio-Technology Co., Ltd. (Shanghai, China; catalog no. B20564; purity >98%, as specified by the supplier). The stock solution was stored at −20 °C. The working vehicle contained 2% DMSO, 40% PEG400, 5% Tween-80, and 53% saline, and the AS-IV injection volume was 10 mL/kg. This formulation was used to solubilize AS-IV for intraperitoneal administration. Sham and TBI control mice received the same drug-free vehicle at the same volume, route, and treatment schedule. During antagonist treatment, groups not receiving MK-801 or TCN-201 received corresponding PBS injections under the same conditions. Nine mice were allocated to each group, of which six were used for behavioral assessments. All nine mice were subsequently used across the downstream tissue-based experiments, with three independent mice per group analyzed in each individual assay as biological replicates. At day 28, mice were deeply anesthetized with 2% sodium pentobarbital (50 mg/kg, i.p.) before perfusion or tissue collection according to the requirements of each assay.

### 2.3 Traumatic brain injury model

An *in vivo* controlled cortical impact (CCI) model was used to induce TBI. Mice were anesthetized with isoflurane (3% for induction and 2% for maintenance), and moderate CCI was produced using a microprocessor-controlled pneumatic impact device (RWD Life Science, Shenzhen, China). A 4-mm-diameter craniotomy was performed between the bregma and lambda sutures on the right side of the skull to expose the dura mater. The 3-mm impactor tip struck the exposed dura at a velocity of 2.5 m/s, a depth of 1.5 mm, and a dwell time of 100 ms (Zhang et al., 2022). Sham-operated mice underwent the same procedure, including craniotomy and dural exposure, but without cortical impact. After surgery, hemostasis was achieved with a gelatin sponge, the incision was closed, and the mice were maintained on a heating blanket at 37 °C until recovery from anesthesia.

### 2.4 Integrated computational and experimental methodology

The human GluN1/GluN2A ligand-binding domain complex (PDB ID: 5H8F) was obtained from the RCSB Protein Data Bank. The structure of AS-IV was retrieved from the PubChem database (CID: 13943297), and molecular docking was performed using Glide to predict a potential binding mode within the receptor complex. Molecular dynamics (MD) simulations were subsequently performed using GROMACS 2020 with the CHARMM36m force field to evaluate the stability and dynamic interactions of the docked AS-IV–GluN1/GluN2A complex. The complex was solvated using the TIP3P water model, neutralized, and adjusted to 150 mM NaCl. After energy minimization, the system was equilibrated under NVT and NPT conditions. A 100-ns production simulation was conducted under NPT conditions at 300 K and 1 bar using a 2-fs time step. The trajectory was analyzed for root-mean-square deviation, root-mean-square fluctuation, radius of gyration, solvent-accessible surface area, and hydrogen-bond formation. Structural visualization was performed using PyMOL. Possible target engagement was further explored using the cellular thermal shift assay (CETSA). HT-22 cells (TCM-C821; FuHeng Biology) were maintained in high-glucose DMEM containing 10% fetal bovine serum and 1% penicillin–streptomycin at 37 °C in 5% CO2. Cells were treated with AS-IV (40 μg/mL) for 6 h and then exposed to a temperature gradient of 37 °C–67 °C, with heating for 2 min at each designated temperature. After thermal treatment, the soluble protein fraction was collected and NMDAR2A levels were determined by immunoblotting. Band intensities at each temperature were normalized to the corresponding 37 °C signal within the same treatment group. Three independent cell-culture experiments were performed (n = 3). AS-IV-associated thermal stabilization was interpreted as exploratory evidence consistent with possible receptor engagement; it does not establish direct binding, binding affinity, receptor activation, or *in vivo* brain target engagement.

### 2.5 Protein extraction and western blotting

Tissue for Western blotting was obtained primarily from the ipsilateral peri-lesional cortex. Three independent mice per group were analyzed; tissue from each mouse constituted a separate biological sample. Tissue was homogenized in RIPA buffer (R0010, Solarbio, Beijing, China) containing protease inhibitors, incubated for 30 min, and centrifuged at 12,000 × g for 15 min at 4 °C. Supernatant protein concentrations were measured using a BCA kit (PC0020, Solarbio). Equal amounts of protein were separated by SDS-PAGE and transferred to PVDF membranes (Millipore, Billerica, MA, USA). Membranes were blocked with 5% non-fat milk for 2 h, incubated with primary antibodies overnight at 4 °C (Additional Table 1), washed with TBST, and incubated with secondary antibodies (ZSGB-BIO, China) for 2 h at room temperature. Signals were detected by enhanced chemiluminescence (Proteintech) and quantified using ImageJ. Each animal contributed one biological data point, irrespective of repeated measurements of its sample.

**TABLE 1:** Antibodies used in the study.

Antibody	Host	Dilution	Application	Supplier	Cat. no.	RRID
Primary antibodies						
NMDAR2A	Rabbit	1:1000 (WB); 1:100 (IF)	WB, IF	Abcam	ab124913	AB_10975154
NMDAR2B	Rabbit	1:2000	WB	Abcam	ab254356	AB_2895296
CaMKII	Rabbit	1:1000	WB	Proteintech	12666-2-AP	AB_2068213
Phospho-CaMKII	Rabbit	1:1000	WB	Cell signaling technology	#12716	AB_2713889
ERK1/2	Rabbit	1:1000	WB	Proteintech	66192-1-Ig	AB_2811205
Phospho-ERK1/2	Rabbit	1:4000	WB	Cell signaling technology	#4370	AB_2315112
CREB	Rabbit	1:1000	WB	Proteintech	67927-1-Ig	AB_2918679
Phospho-CREB (Ser133)	Rabbit	1:2000	WB	Cell signaling technology	#9198	AB_2561044
PSD-95 (WB/single-label IF)	Rabbit	1:1000 (WB); 1:200 (IF)	WB, IF	Proteintech	20665-1-AP	AB_2687961
ARC	Rabbit	1:1000	WB	Proteintech	66550-1-Ig	AB_2881912
SYN	Rabbit	1:1000	WB	Proteintech	17785-1-AP	AB_2271365
BDNF	Rabbit	1:1000	WB	Cell signaling technology	#47808	AB_2108614
MAP-2	Rabbit	1:200	IF	Proteintech	17490-1-AP	AB_2137880
NeuN	Rabbit	1:100	IF	Abcam	ab177487	AB_2532109
BrdU	Mouse	1:50	IF	Proteintech	66241-1-Ig	AB_2881630
PSD-95 (double-label IF)	Mouse	1:100	IF	Cell signaling technology	36233	AB_2721262
Secondary antibodies						
Alexa Fluor® 488 goat anti-mouse IgG	Goat	1:200	IF	ZSGB-BIO ZS	ZF-0512	AB_3662677
Alexa Fluor® 594 goat anti-rabbit IgG	Goat	1:200	IF	ZSGB-BIO ZS	ZF-0516	AB_2936330
HRP-conjugated goat anti-rabbit IgG (H + L)	Goat	1:10,000	WB	Abcam	ab6721	AB_955477

### 2.6 Brain tissue preparation and immunofluorescence analysis

Three independent mice per group were used for each immunofluorescence analysis. Animals were deeply anesthetized with 2% sodium pentobarbital (50 mg/kg, intraperitoneally) and transcardially perfused with ice-cold PBS followed by 4% paraformaldehyde (PFA). Brains were collected, post-fixed overnight in 4% PFA at 4 °C, and cryoprotected sequentially in 20%, 25%, and 30% sucrose. Except for NeuN/BrdU staining described below, frozen coronal sections were cut at 30 μm using a cryostat (Leica CM1950, Germany) at −20 °C. Sections were washed with PBS, blocked for 1 h with 1% BSA and 10% normal goat serum in 0.1% TBS–Triton X-100, and incubated with primary antibodies overnight at 4 °C (Additional Table 1). After washing, species-appropriate Alexa Fluor-conjugated secondary antibodies were applied for 1 h at 25 °C, followed by DAPI counterstaining. For NMDAR2A/PSD-95 double immunofluorescence, rabbit anti-NMDAR2A and mouse monoclonal anti-PSD95 (Cell Signaling Technology, #36233) primary antibodies were used with species-specific fluorescent secondary antibodies. Images were acquired using a Zeiss Axio Imager Z2 microscope and analyzed in ImageJ.

For NeuN/BrdU analysis, BrdU was administered intraperitoneally at 50 mg/kg every other day on post-injury days 1, 3, 5, 7, 9, 11, and 13. Mice were sacrificed on day 28. NeuN/BrdU double immunofluorescence was performed on 10-μm frozen coronal sections from anatomically matched ipsilateral injured/peri-lesional cortex. DNA was denatured with 2 M HCl at 37 °C for 30 min, and anti-BrdU antibody (Proteintech, 66241-1-Ig) was used at 1:50. Three independent mice per group were analyzed.

Immunofluorescence analyses were performed on anatomically matched coronal sections at comparable rostrocaudal levels. Cortical regions of interest were located in the ipsilateral injured/peri-lesional cortex, whereas hippocampal MAP-2 analysis was performed in the dentate gyrus. Images were acquired using identical microscope settings across groups, and quantitative analyses were performed blinded to group allocation.

### 2.7 Modified neurological severity score (mNSS) assessment

The investigators were blinded to the experimental groups during all assessments. Tests were performed in a quiet room maintained at 20 °C ± 2 °C. The modified neurological severity score (mNSS) was used to evaluate motor, sensory, balance, and reflex functions on a scale ranging from 0 (normal) to 18 (maximal deficit) (Chen et al., 2001). Two blinded evaluators assessed neurological deficits 1 day before TBI and on days 1, 3, 7, and 14 after TBI.

### 2.8 Novel object recognition test

The novel object recognition (NOR) test was performed on post-injury day 28 to evaluate non-spatial recognition memory. Mice were habituated to the open-field box for 30 min per day for 3 consecutive days before testing. During the training phase, each mouse was allowed to explore two identical objects for 5 min. After 1 h, one familiar object was replaced with a novel object of a different shape and color. The apparatus and objects were cleaned with 75% ethanol between trials to eliminate residual odors. The test phase lasted 5 min. Exploration times for the familiar object (F) and novel object (N) were recorded. The discrimination ratio was calculated as DR = N/(N + F) × 100% and is expressed as a percentage, whereas the recognition index was calculated as RI = (N − F)/(N + F) and is dimensionless (Ennaceur et al., 1997). Behavior was recorded and analyzed using a tracking system (Shanghai Xinruan, China).

### 2.9 Morris water maze test

The Morris water maze (MWM) was used to assess spatial learning and memory (Morris et al., 1982). The test was conducted in a circular pool (1.2 m in diameter and 40 cm in height) filled with opaque water maintained at 22 °C ± 1 °C. The escape platform was placed in the fourth quadrant. From post-injury days 22–26, mice underwent 5 consecutive days of acquisition training with four 60-s trials per day, and escape latency was recorded. For inferential analysis, the five daily acquisition values were averaged for each mouse to generate one mean escape-latency value per animal. On post-injury day 27, the platform was removed for the probe trial and each mouse was allowed to swim freely for 60 s. The NOR test was conducted on post-injury day 28. All MWM trials were recorded using an overhead camera and analyzed with the ANY-maze 7.0 tracking system. The order of testing was randomized, and investigators were blinded to group allocation.

### 2.10 Golgi-Cox staining

Mice were deeply anesthetized with 2% sodium pentobarbital (50 mg/kg, i.p.) and euthanized by rapid decapitation. The brains were removed and immersed in 5% Golgi-Cox solution, which was replaced after 2 days, followed by incubation in the dark for a total of 14 days. Brain tissues were then sequentially transferred to 20%, 25%, and 30% sucrose solutions. Coronal sections (100 μm) were prepared using a vibrating microtome (Leica, Germany) and mounted on adhesive microscope slides. Sections were rinsed with distilled water, treated with ammonium hydroxide for 30 min, incubated in 5% sodium thiosulfate, dehydrated through graded ethanol, cleared in a chloroform/xylene/absolute ethanol mixture (1:1:1), and mounted with DPX. Images were acquired using a digital slide scanner (Zhong et al., 2019). Three mice per group were analyzed in the ipsilateral injured/peri-lesional cortex. Morphologically intact secondary or tertiary dendrites were selected for spine analysis. Mushroom spines were defined by a distinct neck and enlarged head, thin spines by a slender neck and small head, and stubby spines by a short broad protrusion without a distinct neck. Total spine density was expressed as the number of spines per 10 μm of dendrite.

### 2.11 Transmission electron microscopy

Neuronal ultrastructure was examined by transmission electron microscopy (TEM). Mice were deeply anesthetized with 2% sodium pentobarbital (50 mg/kg, i.p.), and tissue from the ipsilateral cortex and hippocampal CA1 region was rapidly collected and dissected into approximately 1-mm^3^ blocks. Samples were fixed overnight in 2.5% glutaraldehyde at 4 °C, post-fixed with 1% osmium tetroxide for 1 h, dehydrated through graded ethanol and acetone, and embedded in Epon 812 resin. Ultrathin sections (∼70 nm) were prepared, stained with uranyl acetate and lead citrate, and examined using an HT7800 transmission electron microscope (Hitachi, Japan). Three mice per group were analyzed. For each mouse, three tissue blocks were sampled, with three ultrathin sections obtained from each block and six non-overlapping fields examined per section. Images were acquired at approximately 7,000×–3×0,000 magnification.

### 2.12 Statistical analysis

Statistical analyses were performed using SPSS 16.0. Data are presented as mean ± SEM, with p < 0.05 considered statistically significant. Behavioral analyses used six mice per group, whereas tissue-based assays used three mice per group, with each mouse treated as one biological replicate. Repeated measurements from the same animal were averaged to generate one biological value per animal. Normality and homogeneity of variance were assessed using the Shapiro–Wilk and Levene tests, respectively. Group comparisons were performed using one-way ANOVA followed, where appropriate, by LSD or Student–Newman–Keuls (SNK) *post hoc* tests. mNSS scores were analyzed separately at each time point. For MWM acquisition, the five-day escape-latency values were averaged for each mouse for group comparison, whereas daily values were used to illustrate the learning trajectory. Probe-trial and NOR outcomes were analyzed separately using one-way ANOVA.

## 3 Results

### 3.1 AS-IV mitigates TBI-induced recognition memory impairments and enhances neurological function recovery after TBI

First, we evaluated neurological deficits in TBI mice using the modified Neurological Severity Score (mNSS). As shown in Figure 1D, mice exhibited a sharp increase in neurological deficit scores on day 1 post-TBI compared with the sham group. No significant differences were observed among the TBI groups at this time point (p > 0.05). On days 7 and 14, the vehicle-treated TBI group showed slight reductions in mNSS scores. In contrast, the AS-IV- and GM1-treated groups displayed greater decreases, and no significant differences from the sham group were observed in these treatment groups on day 14 (Figure 1D).

To investigate whether AS-IV could ameliorate CCI-induced learning and memory deficits, MWM acquisition training was conducted on post-injury days 22–26, followed by the probe trial on day 27 and NOR testing on day 28. During MWM acquisition, sham mice showed shorter escape latencies than TBI mice, whereas AS-IV-treated mice, particularly the 40 mg/kg group, showed reduced latency relative to the TBI group (Figures 1C,E). In the probe trial, TBI mice showed impaired spatial memory, including fewer crossings of the former platform location and reduced target-quadrant occupancy, while AS-IV treatment improved these measures (Figures 1F,G,I). NOR testing likewise showed impaired recognition memory after TBI and higher discrimination and recognition indices after AS-IV treatment (Figures 1H,J,K). Across several behavioral outcomes, the 40 mg/kg AS-IV group showed greater effects than the 80 mg/kg group.

### 3.2 AS-IV alleviates TBI-induced neuropathological changes

We analyzed microtubule-associated protein 2 (MAP-2) expression and dendritic morphology using immunofluorescence (IF). Statistical analysis showed significant differences between groups in cortical MAP-2 fluorescence intensity in the peri-impact region, with the TBI group showing markedly lower intensity than sham controls (p < 0.01), along with fragmented dendritic morphology (Figures 2A,C). Both 40 mg/kg and 80 mg/kg AS-IV attenuated this reduction, with more preserved dendritic structures observed after treatment. Concurrently, PSD-95, a synaptic scaffolding protein, showed significantly decreased cortical fluorescence intensity after injury (Figures 2B,D). AS-IV-treated groups exhibited higher relative PSD-95 fluorescence intensity than vehicle-treated TBI mice. Notably, the 40 mg/kg dose of AS-IV produced higher relative PSD-95 fluorescence intensity than 80 mg/kg (p < 0.01).

**FIGURE 2:**
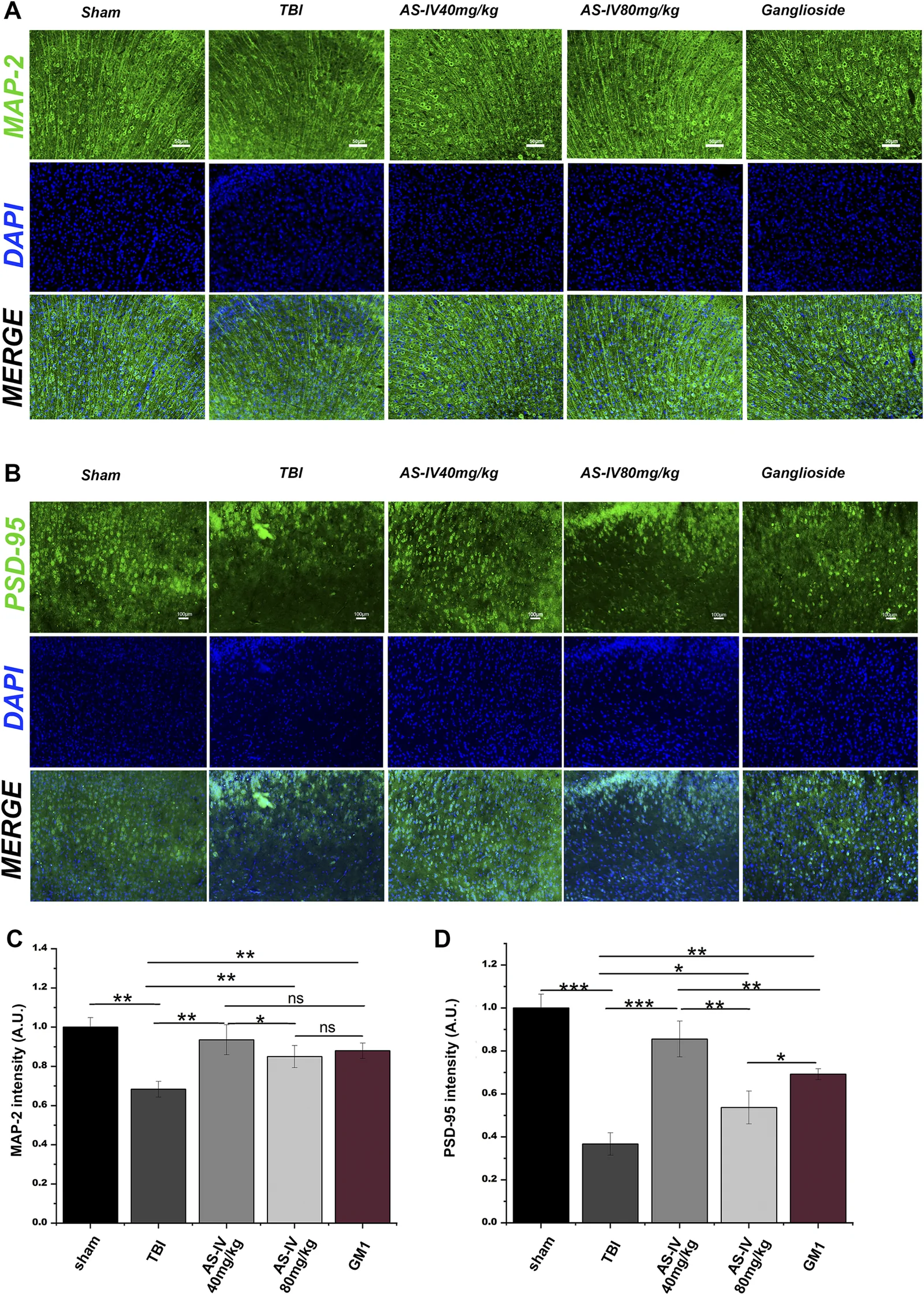
AS-IV preserves MAP-2 and promotes its recovery after TBI. **(A)** Immunofluorescence staining of MAP-2 in cortical neurons (scale bar: 50 μm; n = 3). **(B)** Immunofluorescence staining of PSD-95 in cortical neurons (scale bar: 100 μm; n = 3). **(C)** Quantification of relative cortical MAP-2 fluorescence intensity. **(D)** Quantification of relative cortical PSD-95 fluorescence intensity. Data are mean ± SEM; n = 3 mice per group. *p < 0.05, **p < 0.01, ***p < 0.001; ns, not significant. Sham and TBI groups received the matched drug-free vehicle.

### 3.3 AS-IV treatment is associated with NMDAR subunit expression and related signaling changes after TBI

Western blotting showed significantly lower levels of NMDAR2A and NMDAR2B in TBI mice than in the sham group (Figure 3D). In contrast, AS-IV-treated mice exhibited significantly higher expression of NMDAR2A and NMDAR2B than vehicle-treated TBI mice (Figures 3E,F). Molecular docking was used to evaluate a potential interaction between AS-IV and the human GluN1/GluN2A ligand-binding domain complex. Within the same scoring framework, the docking score of AS-IV suggested a computationally plausible interaction within the receptor complex (Figure 3A). To characterize the structural dynamics of the predicted complex, a 100-ns molecular dynamics simulation was performed. Comprehensive analysis (Figure 3B) showed that the system stabilized after an initial conformational adjustment at approximately 30–35 ns, with backbone RMSD reaching a plateau of 0.32–0.39 nm. The protein maintained overall compactness, as indicated by stable radius-of-gyration and solvent-accessible-surface-area values. Flexibility was mainly localized to terminal and loop regions (residues 40–55 and 150–200), whereas the structural core remained relatively stable. Intermolecular interactions were dynamic, with hydrogen-bond counts generally ranging from 0 to 2 and occasional transient increases. Free-energy landscape analysis projected onto the first two principal components identified a predominant stable conformational state together with interconvertible substates, consistent with the observed conformational rearrangement. In the CETSA experiment, lysates from AS-IV-treated HT-22 cells showed a rightward shift in the thermal denaturation profile of NMDAR2A relative to the vehicle control (Figure 3C). This shift indicated AS-IV-associated thermal stabilization of NMDAR2A in the cellular assay. As shown in Figures 3D–J, during the 28-day recovery period, TBI mice showed significantly reduced ratios of p-ERK1/2 to ERK1/2, p-CaMKII to CaMKII, and p-CREB to CREB compared with sham mice. AS-IV treatment increased these phosphorylation ratios and PSD-95 expression compared with vehicle-treated TBI mice (Figures 3G–J). Differences between the 40 and 80 mg/kg groups varied across the measured molecular endpoints (Figures 3E–J). Collectively, these findings show that AS-IV treatment was associated with changes in NMDAR-related ERK1/2/CaMKII/CREB signaling and increased expression of synapse-associated proteins such as PSD-95, consistent with the behavioral and structural findings.

**FIGURE 3:**
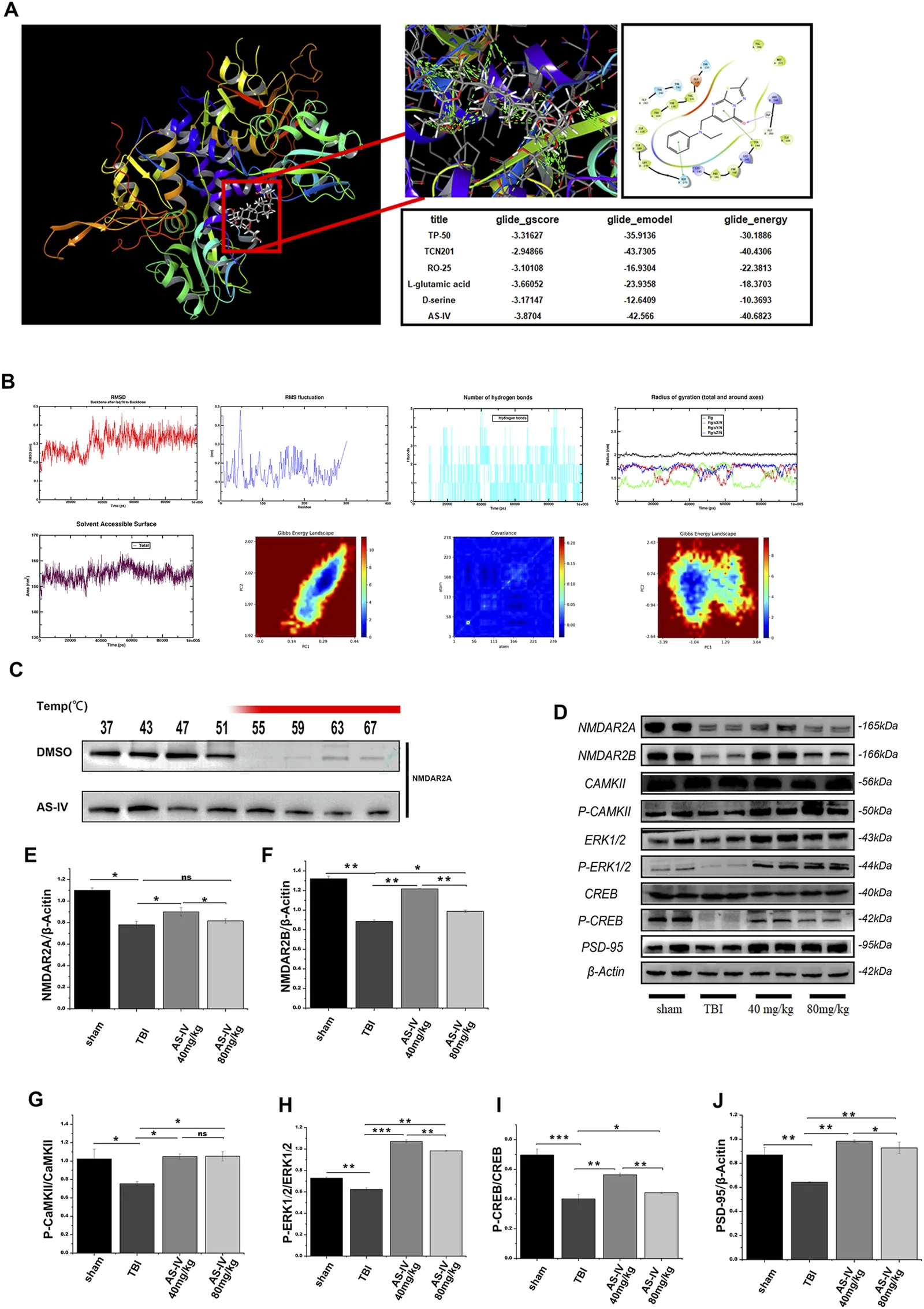
Exploratory receptor-engagement analyses and AS-IV-associated NMDAR-related molecular changes. **(A)** Glide molecular docking of AS-IV to the human GluN1/GluN2A ligand-binding domain complex and comparison of docking scores among the active compounds. **(B)** Structural and dynamic analysis of the AS-IV–GluN1/GluN2A complex during a 100-ns molecular dynamics simulation: (i) backbone RMSD, (ii) per-residue RMSF, (iii) intermolecular hydrogen-bond count, (iv) radius of gyration, (v) solvent-accessible surface area (SASA), (vi) covariance matrix of atomic fluctuations, and (vii) free-energy landscape projected onto the first two principal components. **(C)** CETSA analysis of NMDAR2A thermal stability in HT-22 cells across 37 °C–67 °C; n = 3 independent cell-culture experiments. **(D)** Representative immunoblots of NMDAR-related pathway proteins at 28 days after TBI. **(E–J)** Quantification of NMDAR2A, NMDAR2B, p-CaMKII/CaMKII, p-ERK1/2/ERK1/2, p-CREB/CREB, and PSD-95/β-actin. Animal-derived data are mean ± SEM; n = 3 mice per group for panels **(D–J)**. *p < 0.05, **p < 0.01, ***p < 0.001; ns, not significant. Sham and TBI groups received the matched drug-free vehicle.

### 3.4 NMDAR antagonists attenuate AS-IV-associated cognitive improvement after TBI

To evaluate whether NMDAR-related signaling contributes to the effects of AS-IV, MK-801 and TCN-201 were included in Experiment II (Figure 4A). MWM acquisition was conducted on post-injury days 22–26, followed by the probe trial on day 27 and NOR testing on day 28. The mean escape latency across the five-day MWM acquisition period was higher in the TBI group than in the sham group, reduced by AS-IV monotherapy, and increased again by co-treatment with either TCN-201 or MK-801 (Figure 4D), indicating attenuation of the AS-IV-associated improvement. In the probe trial, AS-IV increased crossings of the former platform location and time spent in the target quadrant compared with TBI controls, whereas both antagonist co-treatment groups showed lower values than the AS-IV monotherapy group (Figures 4E,F). During NOR testing, antagonist co-treatment also attenuated AS-IV-associated improvements in the discrimination ratio and recognition index (Figures 4G–I). Together, antagonist co-treatment attenuated the improvements in MWM and NOR performance observed with AS-IV alone.

**FIGURE 4:**
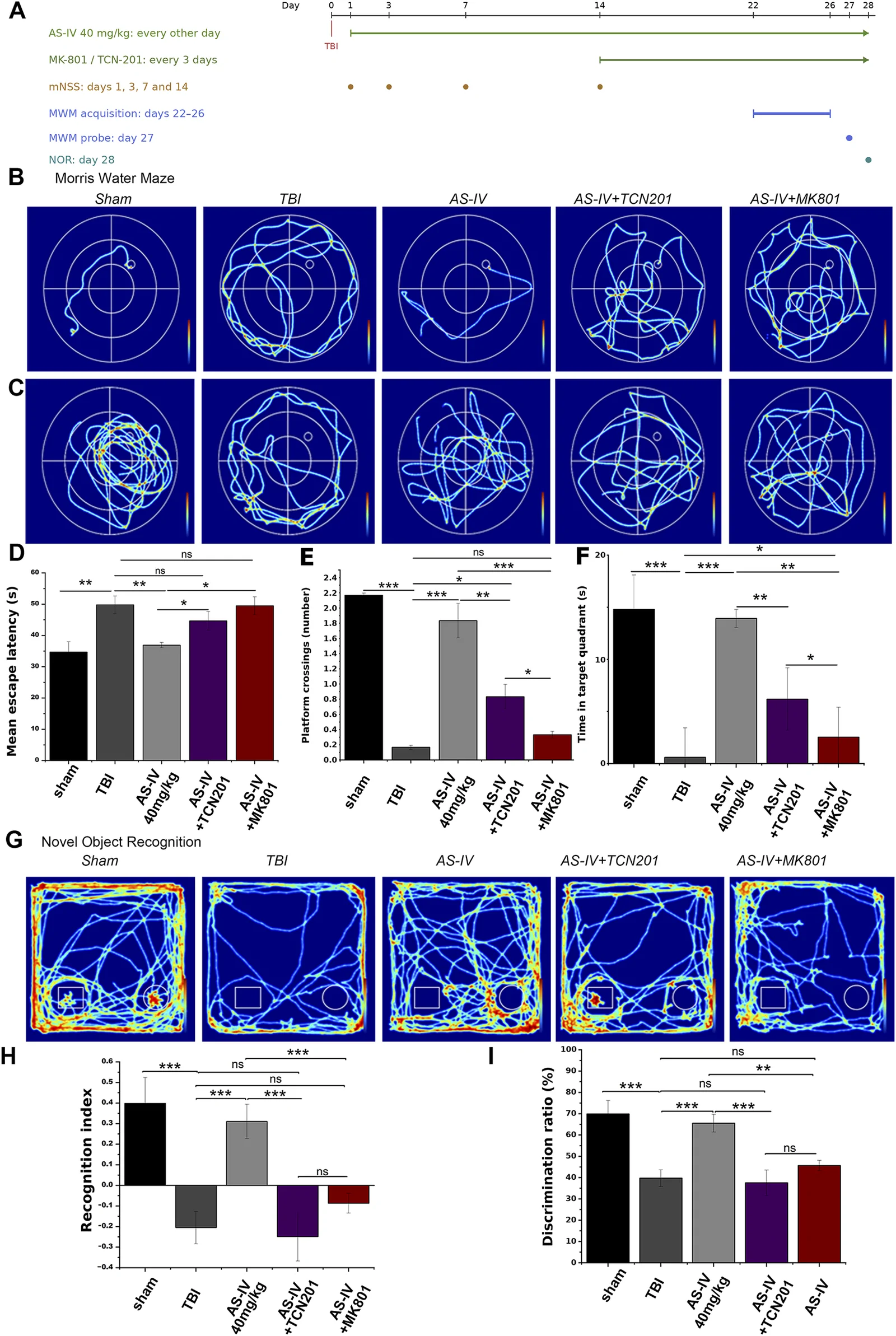
Effects of NMDAR antagonists on AS-IV-mediated cognitive recovery after TBI. **(A)** Timeline of Experiment II. **(B,C)** Representative MWM acquisition trajectories. **(D)** Mean escape latency averaged across the five-day MWM acquisition period (s). **(E)** Number of crossings over the former platform location during the MWM probe trial. **(F)** Time spent in the target quadrant during the MWM probe trial. **(G)** Representative NOR trajectories on day 28 after TBI. **(H)** Recognition index, calculated as (N − F)/(N + F). **(I)** Discrimination ratio, expressed as a percentage [N/(N + F) × 100%]. Data are mean ± SEM; n = 6 mice per group. *p < 0.05, **p < 0.01, ***p < 0.001; ns, not significant. Sham and TBI groups received the matched drug-free vehicle; groups not receiving MK-801 or TCN-201 received corresponding PBS injections during the antagonist-treatment period.

### 3.5 NMDAR antagonist co-treatment attenuates AS-IV-associated preservation of dendritic microtubules

Based on the preceding behavioral findings, we examined histomorphological alterations in mice receiving NMDAR antagonist co-treatment. In the CCI mouse model, cortical neuronal dendrites exhibited pronounced damage in the TBI group compared with the sham group, whereas AS-IV treatment was associated with more preserved dendritic morphology. Immunofluorescence analysis of MAP-2-labeled brain sections revealed that relative MAP-2 fluorescence intensity was significantly reduced in both the AS-IV + MK-801 and AS-IV + TCN-201 groups compared with the AS-IV group (p < 0.01; Figures 5A,C). The AS-IV group demonstrated significantly higher MAP-2 fluorescence intensity than the TBI group (p < 0.001). No significant difference was observed between the two antagonist co-treatment groups. Transmission electron microscopy (TEM) showed that sham-group neurons had distinct nuclear membranes, preserved nuclear structures, and visible mitochondria and Golgi apparatus. Conversely, neurons in CCI-subjected mice displayed disrupted cellular ultrastructure, accompanied by mitochondrial swelling, vacuolization, and cristae disruption (Figure 6A). These pathological features were less pronounced in AS-IV-treated mice. TEM images also revealed fragmented microtubule segments and less organized microtubule arrangements in the TBI group. Neuronal cytoskeletons in both the AS-IV + MK-801 and AS-IV + TCN-201 groups resembled those in the TBI group, exhibiting disorganized and partially disrupted microtubule structures. Consistent with the IF findings, both antagonist co-treatment groups showed less structural preservation than the AS-IV-alone group. Numerous fragmented microtubule structures were visible in the co-treatment groups. In contrast, the AS-IV-alone group showed more intact microtubules within dendritic shafts, with a more organized, parallel bundled arrangement (Figure 6C).

**FIGURE 5:**
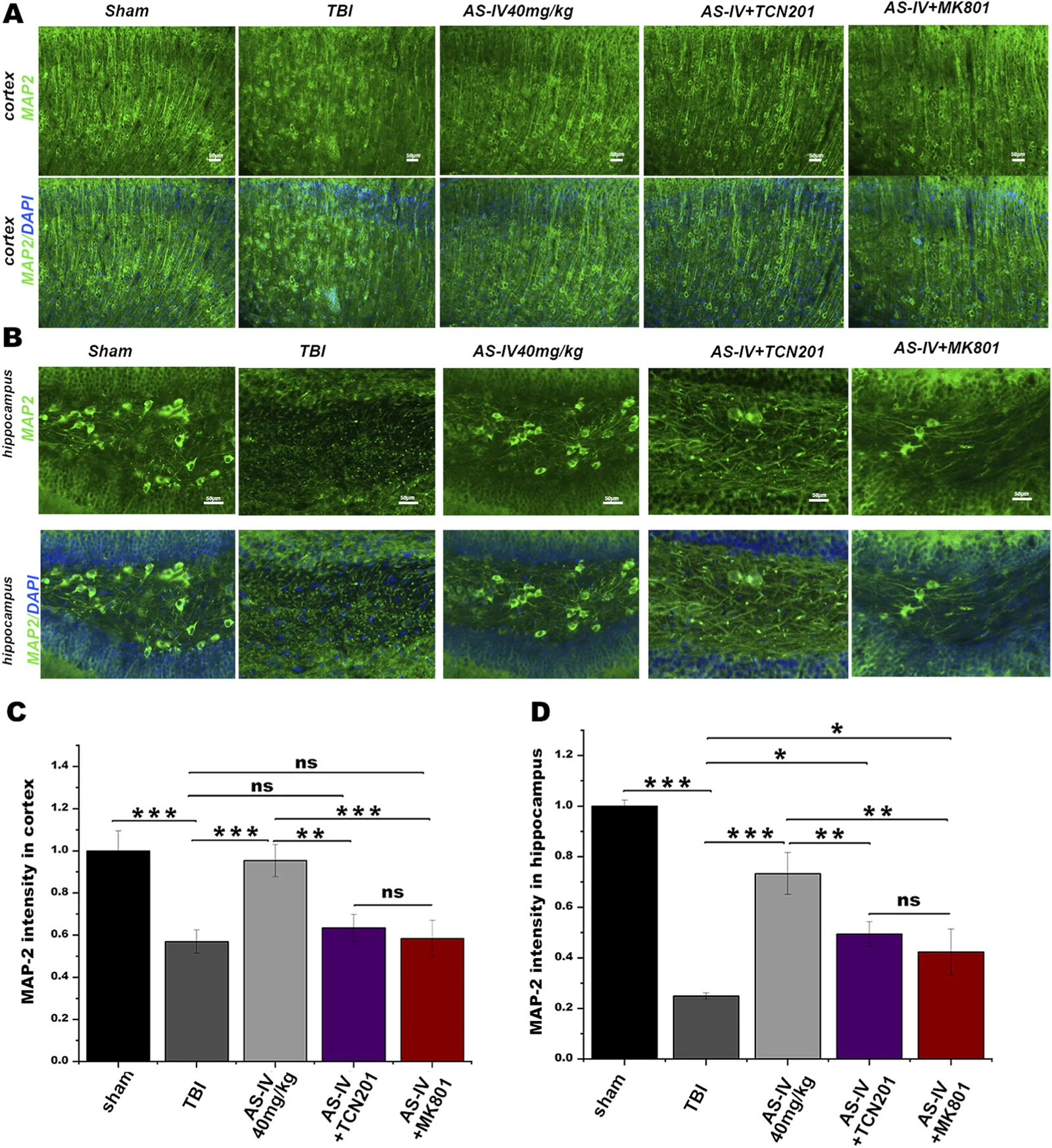
Effects of NMDAR antagonists on AS-IV-mediated MAP-2 recovery in the cortex and hippocampus after TBI. **(A)** Representative MAP-2 immunofluorescence images in the cortex across the five groups (scale bar: 50 μm; n = 3). **(B)** Representative MAP-2 immunofluorescence images in the hippocampal dentate gyrus (DG) across the five groups (scale bar: 50 μm; n = 3). **(C)** Quantification of relative MAP-2 fluorescence intensity in the cortex. **(D)** Quantification of relative MAP-2 fluorescence intensity in the DG. Data are mean ± SEM; n = 3 mice per group. *p < 0.05, **p < 0.01, ***p < 0.001; ns, not significant. Sham and TBI groups received the matched drug-free vehicle; groups not receiving MK-801 or TCN-201 received corresponding PBS injections during the antagonist-treatment period.

**FIGURE 6:**
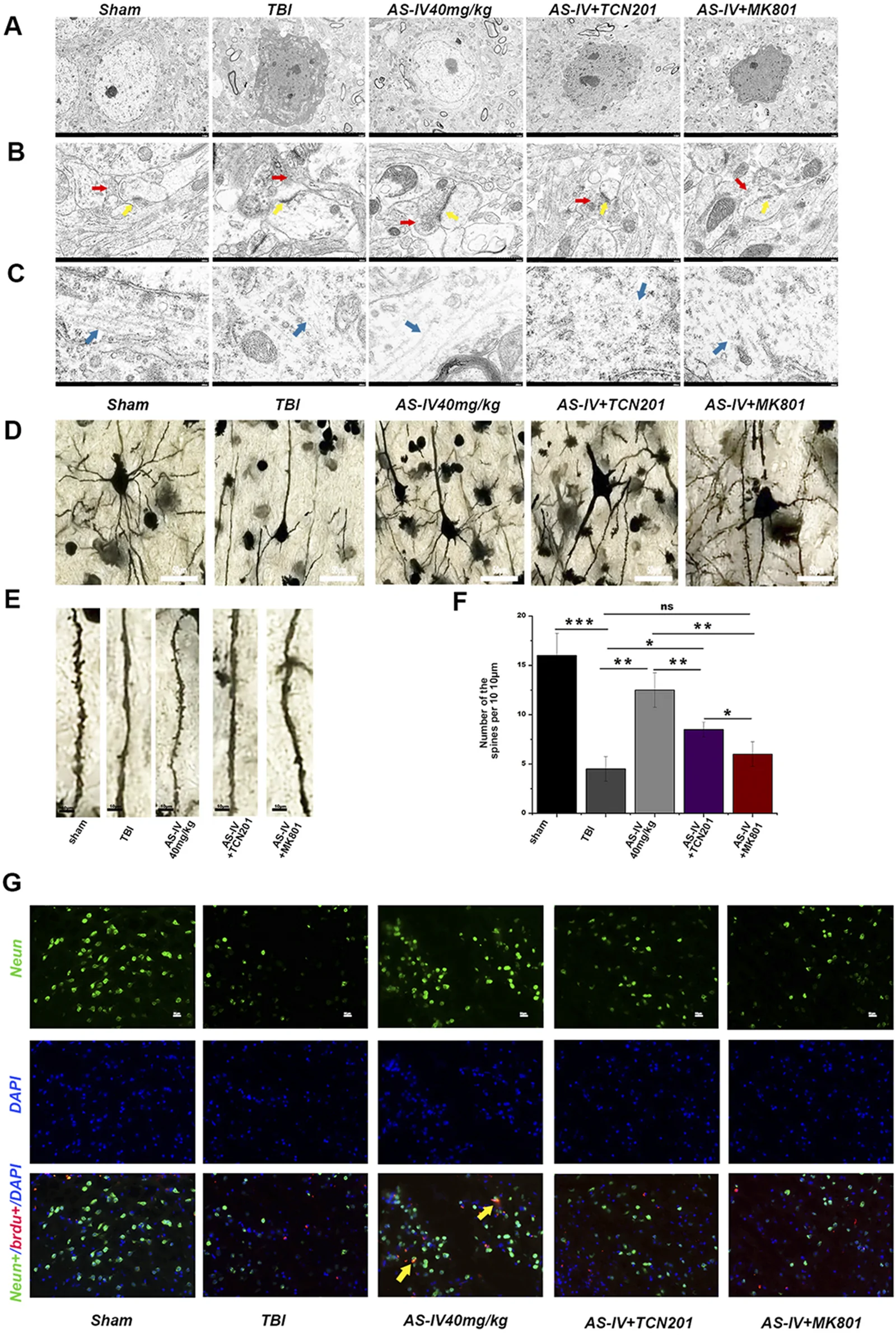
Effects of NMDAR antagonists on AS-IV-mediated recovery of synapses and dendritic spines after TBI. **(A)** Neuronal ultrastructure across the five groups after TBI (scale bar: 5 μm; n = 3). **(B)** Synaptic ultrastructure across the five groups after TBI (scale bar: 5 μm; n = 3). Red arrows indicate synaptic vesicles and yellow arrows indicate the postsynaptic density (PSD). **(C)** Microtubule ultrastructure (scale bar: 5 μm; n = 3); blue arrows indicate microtubules. **(D)** Cortical Golgi-Cox staining showing neuronal morphology (scale bar: 50 μm; n = 3). **(E)** Cortical Golgi-Cox staining showing dendritic spines (scale bar: 10 μm; n = 3). **(F)** Quantification of total dendritic spine density per 10 μm of cortical dendrite. **(G)** Representative NeuN/BrdU double-immunofluorescence images in the injured/peri-lesional cortex (scale bar: 50 μm; n = 3). Data are mean ± SEM; n = 3 mice per group. *p < 0.05, **p < 0.01, ***p < 0.001; ns, not significant. Sham and TBI groups received the matched drug-free vehicle; groups not receiving MK-801 or TCN-201 received corresponding PBS injections during the antagonist-treatment period.

### 3.6 AS-IV treatment is associated with synaptic structural alterations and neuronal markers

To examine structural correlates of behavioral improvement, cortical dendrites were assessed by Golgi-Cox staining. Representative images showed dendritic and spine abnormalities after TBI (Figures 6D,E). Total dendritic spine density was lower in the TBI group and higher in the AS-IV group; both antagonist co-treatment groups showed lower density than the AS-IV-alone group (Figure 6F). Representative images also showed differences in dendritic spine morphology among the experimental groups (Figure 6E).

TEM images showed TBI-associated synaptic ultrastructural abnormalities and a more preserved appearance in the AS-IV group, with less distinct pre- and postsynaptic structures in the antagonist co-treatment groups than in the AS-IV-alone group (Figure 6B). MAP-2 immunoreactivity in the hippocampal dentate gyrus was higher after AS-IV treatment and lower with antagonist co-treatment (Figures 5B,D). NeuN/BrdU-colabeled cells were observed in the ipsilateral injured/peri-lesional cortex at day 28 following BrdU administration on post-injury days 1–13 (Figure 6G). Representative images showed more NeuN/BrdU colabeling in the AS-IV group than in the antagonist co-treatment groups.

### 3.7 NMDAR antagonist co-treatment attenuates AS-IV-associated molecular changes

Given that NMDAR antagonist co-treatment attenuated AS-IV-associated structural changes, we examined whether this pattern was accompanied by changes in NMDAR-related ERK1/2/CaMKII/CREB signaling. As shown in Figure 7A, AS-IV-treated mice had higher NMDAR2A and NMDAR2B expression than TBI mice, whereas both antagonist co-treatment groups showed lower expression (Figures 7B,C). The p-ERK1/2/ERK1/2, p-CaMKII/CaMKII, and p-CREB/CREB ratios were also reduced in the antagonist co-treatment groups (Figures 7D–F). The synapse-associated proteins PSD-95, BDNF, SYN, and ARC showed a similar attenuation pattern (Figures 7G–K). Double immunofluorescence using rabbit anti-NMDAR2A and mouse monoclonal anti-PSD95 (7E3; Cell Signaling Technology, #36233) showed AS-IV-associated increases in the percentage of NMDAR2A/PSD-95 double-positive cells among total cells in the injured cortex, with lower values after MK-801 or TCN-201 co-treatment (Figures 7L,M). Together, these results show that NMDAR antagonist co-treatment attenuated the AS-IV-associated changes in NMDAR subunit expression, ERK1/2/CaMKII/CREB phosphorylation ratios, and synapse-associated molecular markers.

**FIGURE 7:**
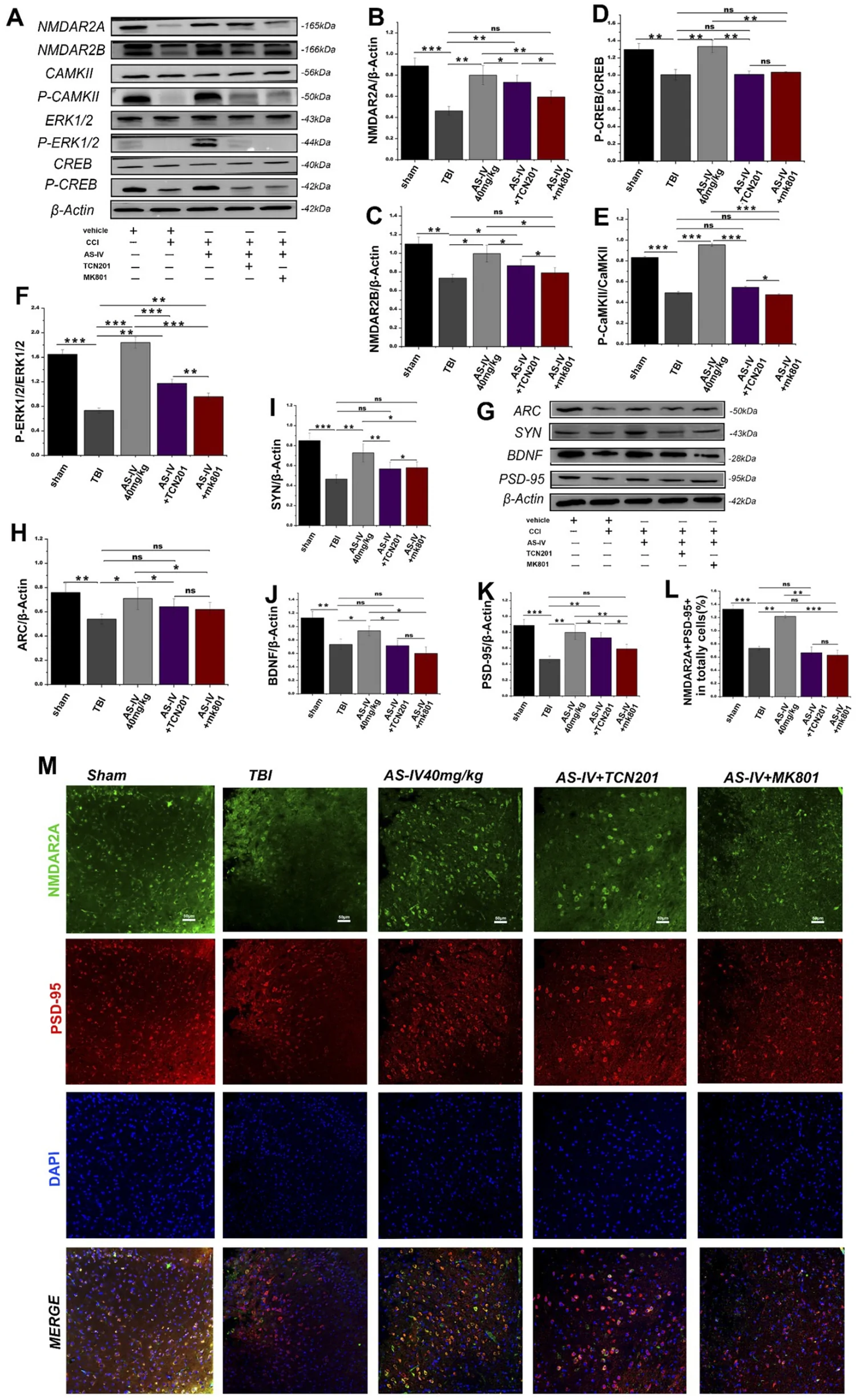
NMDAR antagonist co-treatment attenuates AS-IV-associated signaling and synapse-related molecular changes. **(A)** Representative immunoblots of NMDAR-related pathway proteins at 28 days after TBI. **(B–F)** Quantification of NMDAR2A, NMDAR2B, p-CREB/CREB, p-CaMKII/CaMKII, and p-ERK1/2/ERK1/2. **(G)** Representative immunoblots of synapse-related proteins at 28 days after TBI. **(H–K)** Quantification of ARC, SYN, BDNF, and PSD-95. **(L)** Quantification of NMDAR2A/PSD-95 double-positive cells as a percentage of total cells. **(M)** Representative double-immunofluorescence images of NMDAR2A and PSD-95 in the injured cortex on day 28 after TBI (scale bar: 50 μm; n = 3). Data are mean ± SEM; n = 3 mice per group. *p < 0.05, **p < 0.01, ***p < 0.001; ns, not significant. Sham and TBI groups received the matched drug-free vehicle; groups not receiving MK-801 or TCN-201 received corresponding PBS injections during the antagonist-treatment period.

## 4 Discussion

Traumatic brain injury (TBI), a prevalent global neurological disorder, significantly contributes to worldwide mortality and disability due to neuronal loss and synaptic damage, often manifesting as persistent cognitive dysfunction (Mavroudis et al., 2022). The pursuit of effective therapeutic strategies for TBI management has continued for decades. Current interventions primarily focus on mitigating secondary injury through neuroprotective mechanisms, thereby preserving compromised brain tissue during the early post-injury phase (Tani et al., 2022). Moreover, studies of brain repair, including stroke research, suggest that regenerative approaches during the recovery stage can contribute to functional improvement (Joy and Carmichael, 2021). Experimental and translational research also highlights therapeutic strategies aimed at enhancing motor and cognitive recovery by promoting neurite remodeling and other neurorestorative processes following TBI (Atkinson and Dickman, 2023).

Given the fundamental role of synaptic plasticity in cognitive recovery, exogenous therapeutic interventions may influence synaptic number, structural organization, and functional activity. In alignment with earlier reports of synaptic and signaling abnormalities in experimental TBI (Semchenko et al., 2006; Atkins et al., 2009), our combined analyses using immunofluorescence staining and Western blotting showed notable reductions in PSD-95 expression post-TBI. PSD-95 is a major postsynaptic scaffolding protein involved in postsynaptic organization and signaling (Patel et al., 2019). The relevance of postsynaptic density alterations to TBI pathophysiology has also been discussed previously (Luo et al., 2011). In our study, AS-IV increased PSD-95 expression, accompanied by improved learning and memory performance in the water maze. Alterations in PSD-95 clustering are associated with dendritic spine stability and may therefore be relevant to impaired synaptic organization after injury (Cane et al., 2014). Structural alterations were further examined through Golgi-Cox staining. Dendritic spines are small protrusions extending from neuronal dendrites, and their presence, density, and morphology are closely associated with synaptic organization; spine loss and altered dendritic architecture have been reported after experimental TBI (Winston et al., 2013; Ratliff et al., 2020). Our TBI mice showed a reduction in overall dendritic spine density alongside impaired MWM performance, consistent with the proposed importance of spine remodeling in TBI recovery (Xiong et al., 2019). Related associations between dendritic structure and spatial learning have also been observed in an Alzheimer’s disease mouse model, although that evidence comes from a different disease context (Kan et al., 2018). Morphological plasticity is another essential aspect of spine dynamics. The representative images showed thin, mushroom-shaped, and stubby spine morphologies, which have distinct structural characteristics and remodeling trajectories (Berry and Nedivi, 2017). Only total spine density was quantified in the present study; subtype proportions and numbers were not independently quantified, and static images cannot establish when a spine formed. The AS-IV-associated increase in total spine density therefore supports structural preservation or remodeling rather than demonstrated formation of new functional synapses. Postsynaptic organization and synaptic vesicle distribution also provide information about synaptic structure, as illustrated by studies of postsynaptic complexes and synaptic aging (Zeng et al., 2016; Fan et al., 2018). Here, TEM showed disrupted synaptic organization after TBI, whereas AS-IV-treated tissue exhibited more distinct pre- and postsynaptic structures and more preserved vesicle organization. These observations were qualitative; PSD thickness, vesicle number, and vesicle density were not quantified. Structural and behavioral correlates have also been examined with other compounds in learning-and-memory studies, providing context for the combined approach used here (Luo et al., 2016). Together, PSD-95 measurements, Golgi-Cox staining, and TEM provide complementary evidence for AS-IV-associated synaptic structural and molecular changes. They do not directly measure synaptic transmission, long-term potentiation (LTP), or the establishment of functional connections.

Furthermore, our results showed marked reductions in MAP-2 immunoreactivity and dendritic integrity in the cortex of TBI mice, consistent with the TEM findings. MAP-2, a microtubule-associated protein expressed predominantly in neuronal cell bodies and dendrites, contributes to microtubule organization, neuronal growth, and plasticity (Johnson and Jope, 1992). Its reduced expression therefore suggests disruption of dendritic integrity, as previously reported after traumatic injury (Folkerts et al., 1998; Posmantur et al., 1996). Such structural changes may contribute to persistent cognitive deficits. Neuroinflammation is another potential contributor to cognitive impairment, as illustrated in a lipopolysaccharide model, although its contribution to the present MAP-2 changes was not directly assessed (Zhao et al., 2019). Following AS-IV treatment, fragmented dendritic structures showed a more preserved appearance in the peri-lesional cortex, with higher MAP-2 fluorescence intensity and more organized microtubules in the TEM images. Structural disorganization nevertheless remained in some representative fields. These observations are consistent with attenuation of dendritic injury and possible remodeling, but neurite length, dendritic number, and branching complexity were not separately quantified. Thus, the images support morphological preservation without establishing increases in those specific measures or demonstrating axonal regeneration. Antagonist co-treatment attenuated the AS-IV-associated increases in MAP-2 immunoreactivity and preservation of microtubule organization, linking the dendritic findings to the accompanying behavioral and molecular patterns.

Taken together, AS-IV treatment improved learning and memory performance after TBI. TBI mice treated with AS-IV exhibited shorter escape latencies and greater target-quadrant occupancy in the MWM than vehicle-treated TBI mice. GM1 was included as a positive control because experimental TBI studies have reported effects on axonal outgrowth and cognition (Benady et al., 2018; Rubovitch et al., 2017). The accompanying reduction in neurological deficit scores is consistent with neurological improvement being an important treatment outcome in experimental TBI (Jing et al., 2023). Consistent with the MWM results, TBI mice showed impaired novel-object recognition, whereas AS-IV treatment improved object discrimination. NOR provides a complementary measure of recognition memory rather than a direct measure of attention (Leger et al., 2013). Together, these behavioral findings suggest potential for cognitive recovery, while assessment across multiple time points remains important for characterizing persistent post-TBI deficits (Gold et al., 2013). Notably, 80 mg/kg AS-IV was less effective than 40 mg/kg for several outcomes. The two tested doses do not define a complete dose-response curve or establish an optimal dose. Published AS-IV pharmacokinetic work provides background on systemic disposition in rats (Du et al., 2005), but does not establish exposure in the injured mouse brain under the present intraperitoneal regimen. Because brain and plasma concentrations were not measured, the observed dose pattern cannot be attributed to receptor occupancy, saturable brain entry, or a specific pharmacodynamic mechanism. When TBI mice receiving AS-IV were co-treated with MK-801 or TCN-201, the improvements in NOR and MWM performance were attenuated. Co-treatment did not reproduce the reduction in escape latency observed with AS-IV alone and was associated with lower recognition-memory indices. These results provide pharmacological support for NMDAR-related signaling involvement in AS-IV-associated cognitive recovery, while allowing contributions from additional molecular targets or pathways. Antagonists can themselves affect recognition memory, as demonstrated for MK-801 in other experimental settings (Han et al., 2013). The absence of antagonist-only groups therefore prevents separation of an AS-IV-specific interaction from independent antagonist effects. In addition, a nonsignificant difference between the co-treatment groups does not establish equivalence of their effects or equal receptor inhibition; the present design does not provide a definitive subtype-selective comparison.

The NMDA receptor (NMDAR), situated within the postsynaptic density, responds to glutamatergic neurotransmission and participates in signaling relevant to learning and memory. NMDAR trafficking is important for hippocampal synaptic plasticity and memory consolidation (Yang et al., 2022). Downstream calcium-dependent processes include CaMKII regulation and CREB phosphorylation, which have broad roles in synaptic plasticity (Khan et al., 2021). TBI can impair the capacity for LTP, with time-dependent differences between the total dentate granule-cell population and newly generated granule cells (Weston et al., 2021). Related NMDAR–CREB–BDNF alterations have also been studied in an aging-associated cognitive impairment model (Lian et al., 2021), and NR2B/CaMKII/CREB signaling has been implicated in anesthetic-associated cognitive impairment (Liu et al., 2020; Zhang et al., 2021). These studies provide biological context rather than direct evidence for the mechanism in our CCI model. Following TBI, excessive early glutamatergic signaling can contribute to excitotoxic injury, whereas subsequent changes in NMDAR availability and function may affect recovery (Shohami and Biegon, 2014). Calcium overload and excitotoxicity also feature in other neurological conditions (Yu et al., 2025). The lower GluN2A and GluN2B expression observed here at day 28 is compatible with altered NMDAR-related signaling, but does not identify the processes responsible for receptor loss or establish the preceding time course of receptor activity. In a closed-head-injury mouse model, delayed agonist administration improved recovery in the context of time-dependent NMDAR changes (Biegon et al., 2004). This distinction between early excitotoxicity and later recovery provides a rationale for administering MK-801 and TCN-201 during days 14–28 in the present study. Inhibition during this interval may interfere with signaling that supports activity-dependent remodeling and learning, consistent with attenuation of the behavioral, structural, and molecular changes associated with AS-IV. However, receptor currents and calcium flux were not measured, and our data do not show that AS-IV directly activates NMDARs or that acute NMDAR stimulation would be beneficial. AS-IV-associated increases in the p-CaMKII/CaMKII ratio may reflect modulation of recovery-related signaling, but CaMKII membrane translocation and AMPAR insertion remain untested mechanisms. NMDAR signaling is also mechanistically diverse, including flux-independent processes (Montes de Oca Balderas, 2018), which further limits inference from subunit abundance alone. Previous research has associated TBI with deficits in CREB signaling (Atkins et al., 2009). Our experiment likewise identified a reduction in the p-CREB/CREB ratio in cerebral tissue at 28 days post-injury. CREB-related mechanisms have been examined in therapeutic studies of brain trauma, including sitagliptin-associated neuroprotection and PERK-related regulation of CREB and PSD-95 (DellaValle et al., 2016; Sen et al., 2017). AS-IV treatment increased CREB phosphorylation relative to total CREB, supporting an association between CREB-related signaling and the observed cognitive improvement. These concurrent molecular changes are compatible with NMDAR-related pathway involvement, but do not establish a temporally ordered or exclusive NMDAR–CaMKII/ERK–CREB causal cascade.

During recovery, NMDAR-related signaling may influence ERK-associated processes relevant to neuronal remodeling. Studies in cultured hippocampal neurons show that synaptic and extrasynaptic NMDARs can have opposing effects on ERK activity (Ivanov et al., 2006), while neuroplastin-related changes in ERK signaling have been linked to cognitive function in mice (Amuti et al., 2016). Thus, altered ERK phosphorylation cannot by itself specify the location or mode of receptor activity. CREB-related transcription and BDNF expression have been linked in other experimental contexts, including stress-related hippocampal changes (Alboni et al., 2011), and MAPK and CREB contribute to recognition-memory consolidation (Bozon et al., 2003). Previous work in stroke rats also associates ERK/CREB/BDNF signaling with neuroregenerative responses (Rao et al., 2024). Studies of an Astragalus membranaceus–ligustrazine combination in cerebral ischemia-reperfusion injury, BDNF–TrkB/NMDAR interactions in propofol-associated cognitive dysfunction, and tanshinone I in learning-and-memory models provide further context for these interconnected pathways (Tang et al., 2023; Zhou et al., 2018; Kim et al., 2009). In the present study, antagonist co-treatment attenuated the AS-IV-associated changes in ERK1/2 and CREB phosphorylation, synapse-associated proteins, MAP-2-positive structures, and neuronal and synaptic morphology. These findings support involvement of related signaling processes without establishing a single linear mechanism. Exploratory docking and 100-ns molecular dynamics simulations suggested a possible interaction between AS-IV and the human GluN1/GluN2A ligand-binding domain complex, while CETSA showed AS-IV-associated thermal stabilization of NMDAR2A in HT-22 cells. These observations provide supportive evidence consistent with possible engagement, but the CETSA shift does not distinguish direct binding from indirect stabilization. Docking, MD, and CETSA do not establish binding affinity, receptor activation, or *in vivo* target occupancy. In addition, AS-IV-treated tissue showed less neuronal ultrastructural damage and NeuN/BrdU-colabeled cells in the injured/peri-lesional cortex. These observations are consistent with neurogenesis-associated cellular changes. BrdU incorporation reflects DNA synthesis and can also arise from DNA repair or aberrant cell-cycle activity; NeuN colabeling alone cannot establish the generation, maturation, or functional integration of newborn neurons. Studies of neurogenic responses after focal ischemia, Korean red ginseng in mice, curcumin in Alzheimer’s disease mice, and gliptins after CCI illustrate the relevance of examining these processes with complementary endpoints (Song et al., 2014; Ryu et al., 2020; Lou et al., 2024; Hung et al., 2023). In our study, NeuN/BrdU findings were descriptive, and no conclusion about quantified neuronal production can be drawn. Ki67 identifies active cell cycling and DCX identifies immature neuronal populations; neither alone establishes mature neuronal function. Lineage tracing, complementary developmental-stage markers, and electrophysiological assessment would be needed to determine whether the labeled cells represent newly generated neurons that mature and integrate into functional circuits.

This investigation, while providing evidence that AS-IV improves cognitive performance in mice after TBI, has several limitations. First, although the findings support involvement of NMDAR-related ERK1/2/CaMKII/CREB signaling in AS-IV-associated effects, contributions from other molecular targets and parallel pathways cannot be excluded. The potential polypharmacology of AS-IV and the mechanisms regulating NMDAR subunit expression warrant further investigation; direct binding, functional receptor assays, and genetic approaches would help clarify target engagement and causality. Second, the study documented structural and molecular changes in neurons and synapses but did not directly assess electrophysiological function. Future studies should include LTP and miniature excitatory postsynaptic-current recordings, informed by experimental work linking post-TBI cognitive outcomes to postsynaptic transmission (Wang et al., 2023); functional MRI may provide complementary information on network-level recovery (Xiao et al., 2015). Third, the absence of antagonist-only groups limits attribution of the co-treatment effects to interference with an AS-IV-specific mechanism. Fourth, most neuronal analyses were performed at 28 days after injury. This 28-day window does not establish long-term durability, safety, or the effectiveness of delayed treatment initiation. Defining the therapeutic window will require serial assessment across acute, subacute, and longer-term recovery phases. Fifth, the exclusive use of adult male mice is a major limitation. Sex-dependent changes in post-traumatic synaptic signaling have been reported (Svirsky et al., 2024), and the present findings should not be assumed to generalize to females. Future studies should include both sexes and consider hormonal status and treatment-by-sex interactions. Sixth, brain and plasma exposure were not measured, and the two-dose design cannot establish pharmacokinetic or exposure-response relationships. Finally, although the principal analyses focused on the ipsilateral cortex, MAP-2 changes were also observed in the hippocampal dentate gyrus; NeuN/BrdU staining was performed in the injured/peri-lesional cortex and does not establish hippocampal neurogenesis. The relationship between dentate-gyrus function and recognition memory is supported by experimental work on zinc influx and LTP (Suzuki et al., 2015), but the regional contribution to AS-IV-associated recovery remains unresolved. Future region-specific studies incorporating lineage tracing, maturation markers, and functional assessments should determine whether newly generated neurons contribute to cognitive recovery.

## 5 Conclusion

In conclusion, our study demonstrates that astragaloside IV (AS-IV) improves cognitive performance and attenuates neuronal and synaptic structural abnormalities after traumatic brain injury. These beneficial effects were accompanied by changes in NMDAR-related ERK1/2/CaMKII/CREB signaling and increased expression of synapse-associated proteins, while pharmacological inhibition with MK-801 or TCN-201 attenuated several AS-IV-associated effects. Together, these findings support involvement of NMDAR-related signaling in AS-IV-associated recovery after TBI, although additional molecular targets and signaling pathways may also be involved (Figure 8). Our results support further investigation of AS-IV as a potential multi-target therapeutic candidate for post-traumatic cognitive impairment, with particular attention to dose-response relationships, synaptic function, target engagement, and long-term efficacy and safety.

**FIGURE 8:**
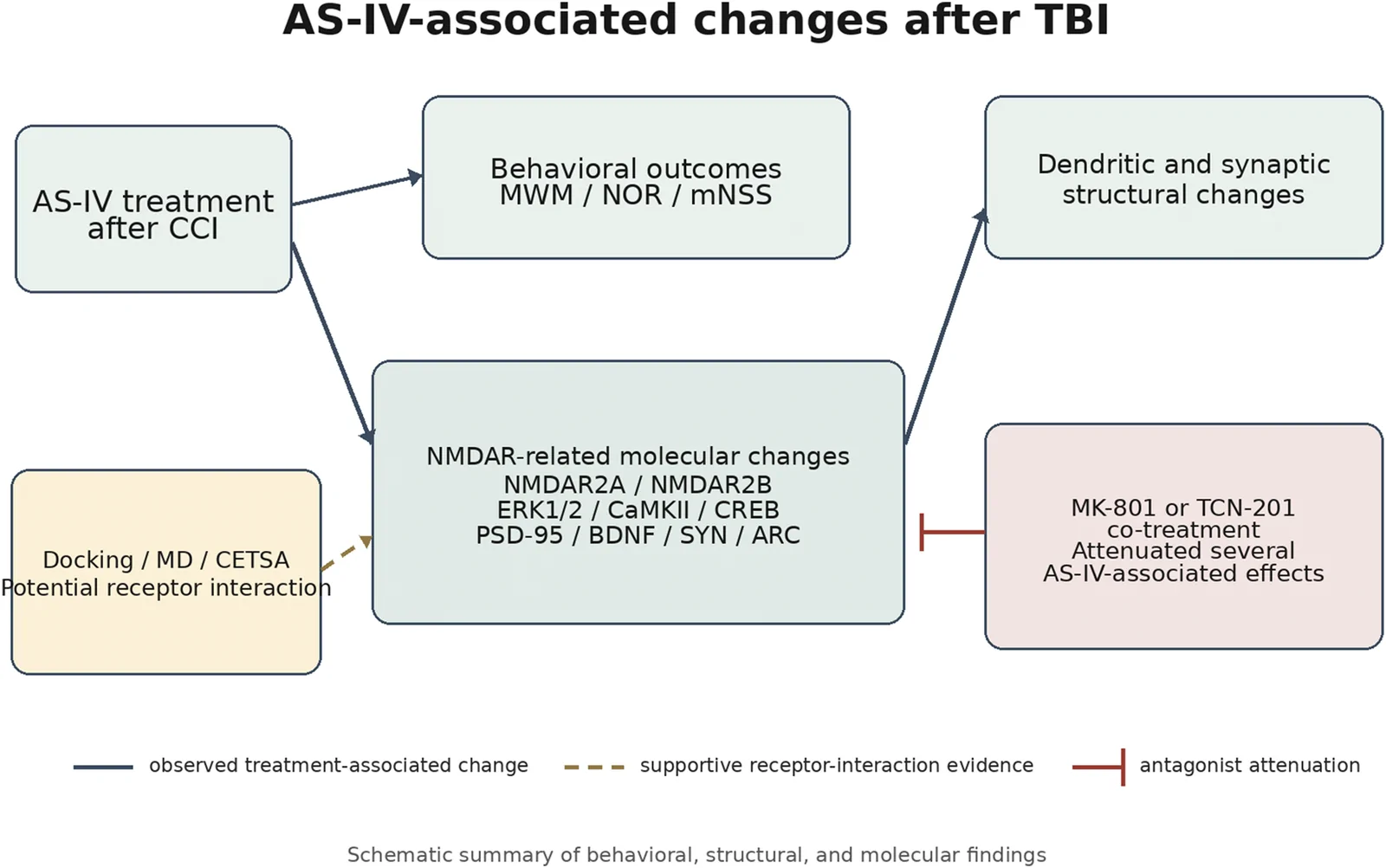
Proposed model of AS-IV-associated NMDAR-related signaling after TBI. Solid arrows indicate observed treatment-associated changes, dashed lines indicate potential receptor interaction supported by docking, molecular dynamics, and CETSA, and the inhibitory line indicates attenuation associated with MK-801 or TCN-201 co-treatment.

## Data Availability

The original contributions presented in the study are included in the article/supplementary material, further inquiries can be directed to the corresponding author.
